# Missing data estimation in extreme rainfall indices for the Metropolitan area of Cali - Colombia: An approach based on artificial neural networks

**DOI:** 10.1016/j.dib.2021.107592

**Published:** 2021-11-19

**Authors:** Camilo Ocampo-Marulanda, Wilmar L. Cerón, Alvaro Avila-Diaz, Teresita Canchala, Wilfredo Alfonso-Morales, Mary T. Kayano, Roger R. Torres

**Affiliations:** a Faculty of Natural Sciences and Engineering, Fundación Universitaria de San Gil, Unisangil, Km 2 via Matepantano, Yopal 850001, Colombia; b Water Resources Engineering and Soil (IREHISA) Research Group, School of Natural Resources and Environmental Engineering, Universidad del Valle, Calle 13 # 100-00, Cali 25360, Colombia; c Department of Geography, Faculty of Humanities, Universidad del Valle, Calle 13 # 100-00, Cali 25360, Colombia; d Universidad de Ciencias Aplicadas y Ambientales - UDCA, Bogota 111166, Colombia; e Natural Resources Institute, Universidade Federal de Itajubá, Itajubá 36570-900, MG, Brazil; f Perception and Intelligent Systems (PSI) Research Group, School of Electrical and Electronics Engineering, Universidad del Valle, Calle 13 # 100-00, Cali 25360, Colombia; g Coordenação Geral de Ciências da Terra, Instituto Nacional de Pesquisas Espaciais, Avenida dos Astronautas, 1758, São José dos Campos, SP 12227-010, Brazil

**Keywords:** Complete missing data, Reconstructs time series, Extreme values of the indices, ETCCDI indices, NLPCA

## Abstract

Changes observed in the current climate and projected for the future significantly concern researchers, decision-makers, and the general public. Climate indices of extreme rainfall events are a trend assessment tool to detect climate variability and change signals, which have an average reliability at least in the short term and given climatic inertia. This paper shows 12 climate indices of extreme rainfall events for annual and seasonal scales for 12 climate stations between 1969 to 2019 in the Metropolitan area of Cali (southwestern Colombia). The construction of the indices starts from daily rainfall time series, which although have between 0.5% and 5.4% of missing data, can affect the estimation of the indices. Here, we propose a methodology to complete missing data of the extreme event indices that model the peaks in the time series. This methodology uses an artificial neural network approach known as Non-Linear Principal Component Analysis (NLPCA). The approach reconstructs the time series by modulating the extreme values of the indices, a fundamental feature when evaluating extreme rainfall events in a region. The accuracy in the indices estimation shows values close to 1 in the Pearson's Correlation Coefficient and in the Bi-weighting Correlation. Moreover, values close to 0 in the percent bias and RMSE-observations standard deviation ratio. The database provided here is an essential input in future evaluation studies of extreme rainfall events in the Metropolitan area of Cali, the third most crucial urban conglomerate in Colombia with more than 3.9 million inhabitants.

## Specifications Table

**Table:** 

Subject	Environmental Science – Climatology
Specific subject area	Missing data estimation in extreme annual and seasonal rainfall indices using an artificial neural network approach.
Type of data	Figures and tables
How the data were acquired	Daily rainfall data were obtained following the procedures established by the Corporación Autónoma Regional del Valle del Cauca (CVC) and the Instituto de Hidrología, Meteorología y Estudios Ambientales (IDEAM) of Colombia.
Data format	Analyzed data Spreadsheet
Description of data collection	The climate rainfall extreme indices were estimated using the ClimInd package, and the indicators are based on daily rainfall (RR) information of surface data. Annual and seasonal extreme rainfall indices were PRCPTOT, RX1day, RX5day, d95p, CWD, CDD, R1mm, R3mm, R10mm, R20mm, and SDII. Missing data estimation in extreme annual and seasonal rainfall indices using an auto-associative neural network known as the Non-Linear Principal Component Analysis approach.
Data source location	Metropolitan Area of Cali, Valle del Cauca–Colombia.
Data accessibility	The daily rainfall dataset is accessible on the CVC: https://ecopedia.cvc.gov.co/portal-hidroclimatologico.html and IDEAM: http://dhime.ideam.gov.co/atencionciudadano/. Extreme rainfall index series are available in this article.

**Value of the Data**

• Data from this article can be used to (a) visualize the relevance of climate risk management studies, (b) improve trend analyses of extreme rainfall events, (c) analyze changes in extreme rainfall indices related to climate variability and change, (d) identify homogeneous climatic regions, and (e) increase the reliability of forecasting in extreme rainfall events.
• The datasets of extreme rainfall indices assess the intensity, frequency, and duration of extreme weather events.
• This dataset can be a proxy for hydrometeorological hazards such as droughts, floods, and heavy rains in the analyzed region.
• The new dataset is useful for institutions, researchers, and experts involved in climate risk management, water resource management, and other fields related to climate variability and change.

## 1 Data Description

**Fig. 1:**
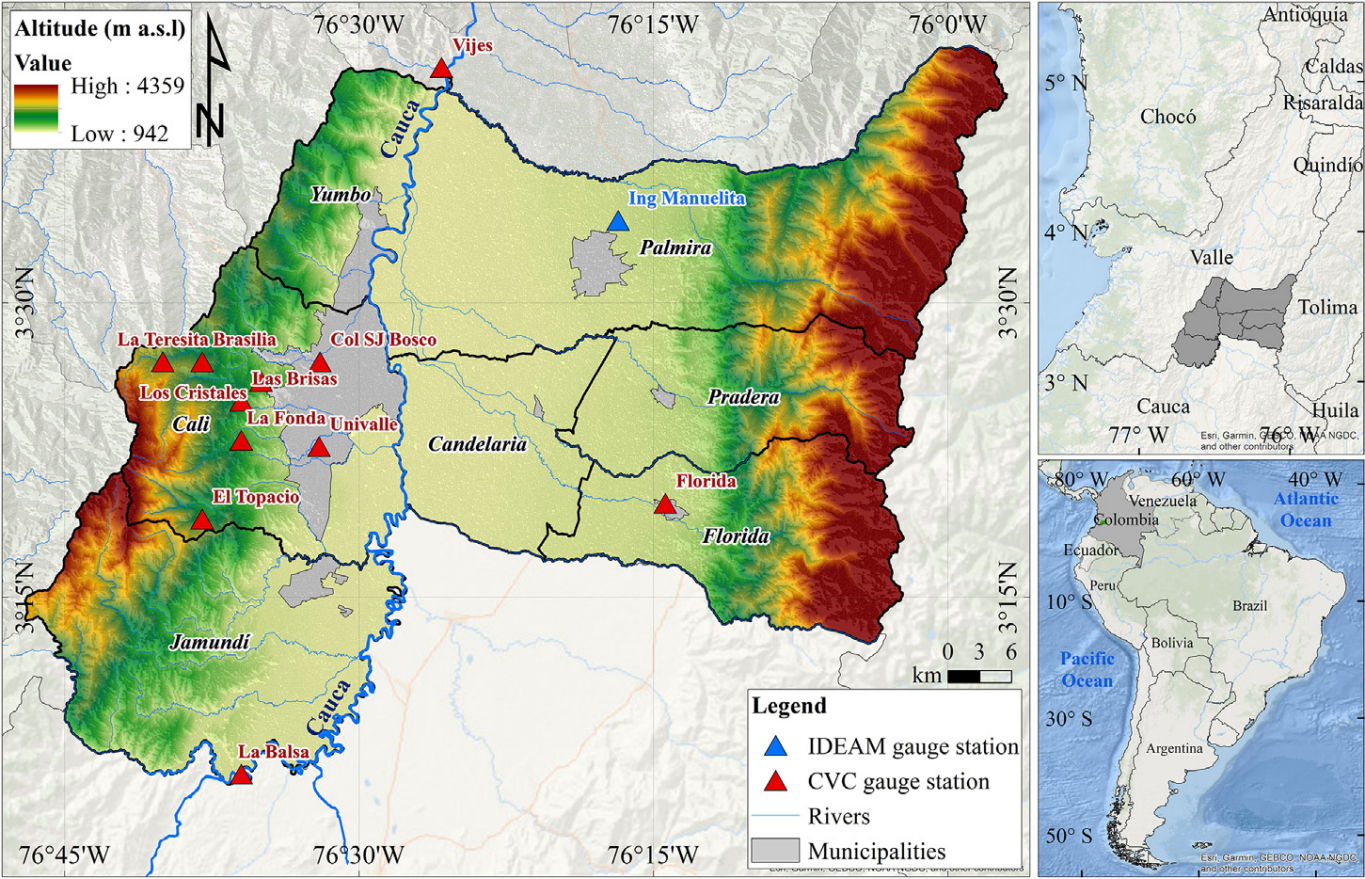
Localization of the rainfall gauge stations in Metropolitan Area of Cali, Valle del Cauca–Colombia.

This paper reports the time series of extreme rainfall indices for the Metropolitan area of Cali in southwestern Colombia - South America (Fig. 1) between 1969 to 2019. Daily rainfall time series from 12 stations were used to construct the extreme rainfall indices. The stations are presented in Fig. 1 and the statistical description of the rainfall data series is given in Table 1. The total annual rainfall for the overall period is between 890.9 and 2803.2 mm, and the standard deviation varies between 211.3 and 542 mm. The mean daily rainfall amounts ranges from 2.5 and 7.7 mm day^–1^, with a standard deviation between 6.7 and 14.1 mm day^–1^ (See Table 1).

**Table 1:** Descriptive statistical analysis of the daily rainfall data in the Metropolitan Area of Cali (1969–2019).

ID	Station	Rainfall mean (mm.year^–1^)	Annual rainfallstandard deviation (mm)	Daily Average(mm)	Daily rainfall standard deviation (mm)
1	Vijes	890.9	258.8	2.5	6.7
2	Ing. Manuelita	1142.1	346.2	3.1	8.0
3	Colegio SJ Bosco	1157.2	455.1	3.3	8.2
4	Univalle	1397.4	211.3	4.0	9.6
5	Los Cristales	1757.7	225.6	4.9	11.2
6	Las Brisas	1966.8	432.5	5.6	12.5
7	Brasilia	1470.9	218.7	4.1	8.5
8	La Teresita	1755.9	380.1	4.9	9.3
9	La Fonda	1948.7	542.0	5.4	12.2
10	El Topacio	2803.2	395.0	7.7	14.1
11	Florida	1544.5	388.1	4.3	11.0
12	La Balsa	2125.8	388.9	5.9	12.2

Twelve rainfall extreme indices were selected, which monitor rainfall intensity (5 indices), frequency (5 indices), and duration (2 indices). The description of the climate indices based on daily rainfall used is presented in Table 2.

**Table 2:** Climatic indices based on the daily rainfall.

Label	Index name	Description	Units
PRCPTOT	Total wet-day rainfall	Rainfall amount on days with RR≥1 mm	mm
RX1day	The highest amount of daily rainfall	The maximum consecutive 1-day rainfall	mm
RX5day	The maximum consecutive 5-day rainfall	The maximum consecutive 5-day rainfall	mm
d95p	Very wet days	Days with rainfall > 95p	days
CWD	Consecutive wet days	The maximum length of consecutive wet days (RR≥1)	days
CDD	Consecutive dry days	The maximum length of consecutive dry days (RR<1 mm)	days
DryDays	Dry Days	Number of days with less than 1 mm	days
R1 mm	Wet days 1 mm	Annual count of wet days	days
R3 mm	Wet days 3 mm	Annual count of days when RR≥ 3 mm	days
R10 mm	Wet days 10 mm	Annual count of days when RR≥ 10 mm	days
R20 mm	Wet days 20 mm	Annual count of days when RR≥ 20 mm	days
SDII	Simple rainfall intensity index	Sum of rainfall in wet days (days with >1 mm of rainfall), and dividing that by the number of wet days in the period	mm.day^−1^

*RR is the daily rainfall

Rainfall stations up to 6% of missing data in the considered period were selected. The daily rainfall series contained missing data, consequently the extreme rainfall index series is also compromised. Due to the criteria adopted in its calculations, the percentage of missing data of the extreme rainfall indices is typically higher than the daily data percentage. Zhang et al. [1] specify that a monthly index is not calculated if more than three daily data are missing in a month, and an annual index is not calculated if more than 15 spread daily data or a month are missing in a year. Table 3 shows the percentage of missing data for the daily precipitation time series and the percentage of missing data for the extreme rainfall indices for annual and seasonal scales.

**Table 3:** Percentage of missing data for daily rainfall data and time scales of extreme rainfall indices.

		Missing data (%)					
ID	Station	Daily rainfall	Annual indices	DJF indices	MAM indices	JJA indices	SON indices
1	Vijes	3.54	13.73	13.73	1.96	5.88	11.76
2	Ing. Manuelita	2.46	7.84	5.88	5.88	3.92	1.96
3	Colegio SJ Bosco	3.00	21.57	39.22	33.33	27.45	41.18
4	Univalle	5.38	27.45	35.29	27.45	39.22	52.94
5	Los Cristales	1.43	15.69	7.84	7.84	11.76	9.80
6	Las Brisas	3.54	17.65	15.69	3.92	11.76	11.76
7	Brasilia	0.82	5.88	9.80	5.88	15.69	7.84
8	La Teresita	3.95	23.53	21.57	17.65	21.57	21.57
9	La Fonda	1.33	13.73	9.80	0.00	3.92	5.88
10	El Topacio	0.53	1.96	3.92	11.76	15.69	5.88
11	Florida	1.04	7.84	1.96	1.96	1.96	9.80
12	La Balsa	0.74	5.88	7.84	1.96	0.00	5.88

**Fig. 2:**
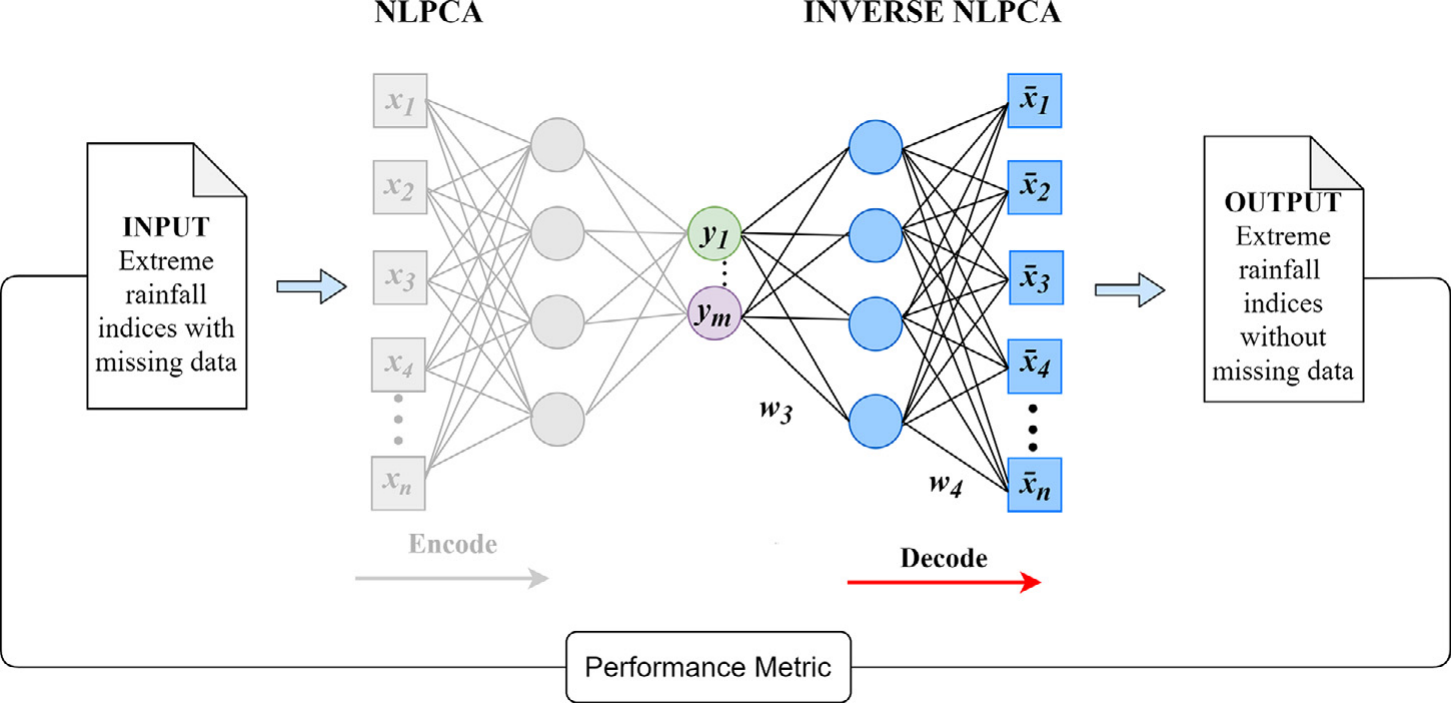
Flowchart of the NLPCA. $x_{n}$ is the input layer (Extreme rainfall indices), $y_{m}$ is the bottleneck layer of the NLPCA model, and ${\bar{x}}_{n}$ is the output layer (Extreme rainfall indices reconstructed).

The missing data of the climate indices were filled using the complete Non-Linear Principal Component Analysis (NLPCA) topology, where the decoder is used after the bottleneck, i.e., the inverse NLPCA, which takes the principal components to recover the original information. Fig. 2 shows the flow chart of the methodology.

**Fig. 3:**
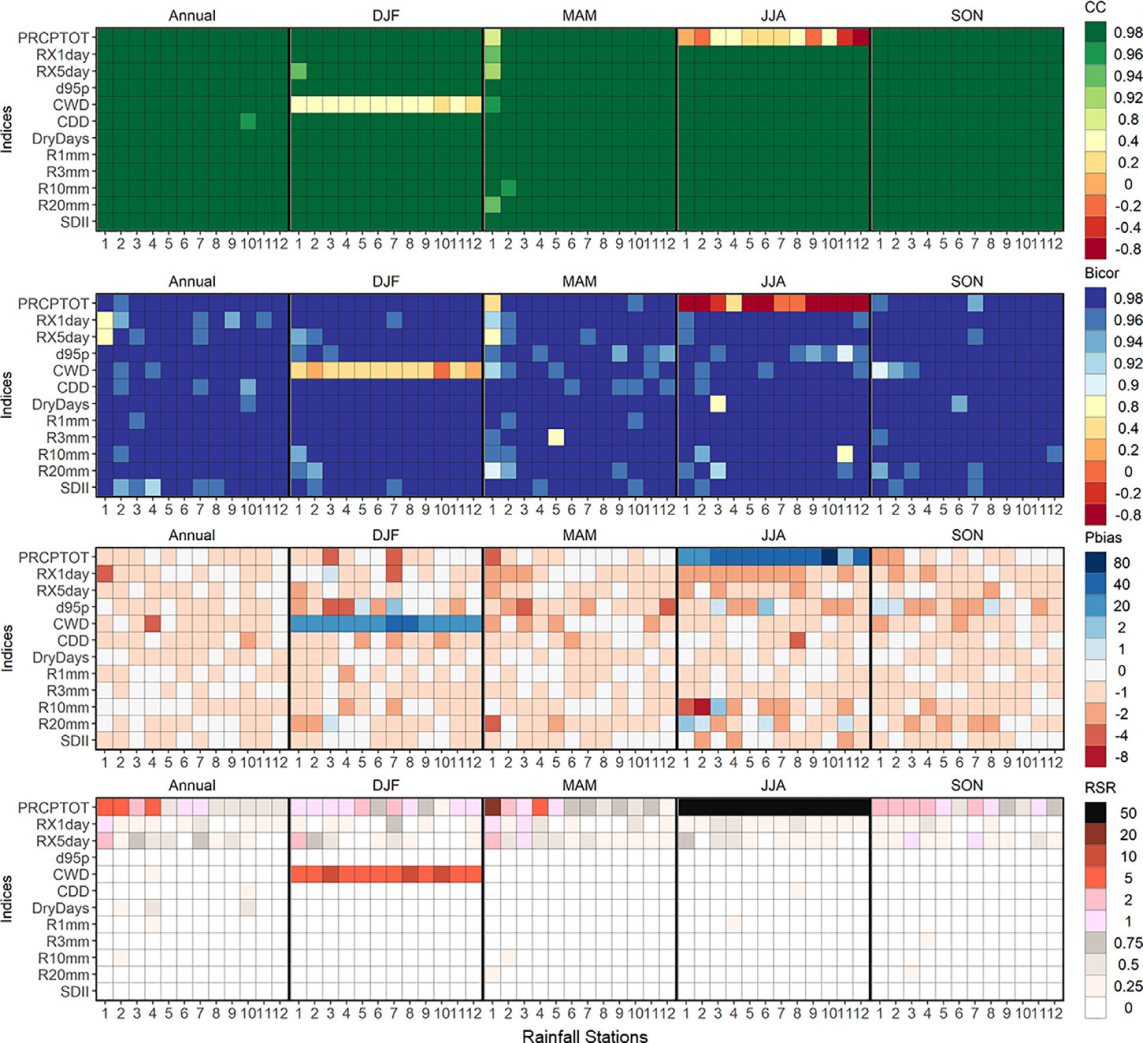
Statistics of missing data estimation error obtained for annual and seasonal extreme rainfall indices from 1969 to 2019 over twelve rainfall stations (Fig. 1). Furthermore, for Pbias and RSR dark (light) colors indicate better (worse) statistical estimation.

Fig. 3 shows the heat map constructed with R v.4.1.2 using ggplot package that representing the performance metrics of extreme precipitation indices at annual and seasonal scales. The assessed through the Pearson's correlation coefficient (CC), Bi-weighting correlation (Bicor), percent bias (Pbias), and RMSE-observations standard deviation ratio (RSR) was estimated. The rows represent the extreme rainfall indices, and the columns represent the stations grouped by the time scales studied. The performance in the estimation of extreme rainfall indices is highlighted with CC and Bicor (Pbias and RSR) values close to 1 (0) in the all-time series, except for CWD and PRCPTOT during December to February (DJF) and June to August (JJA), respectively. The equations for CC, Bicor, Pbias and RSR are shown in Table 4.

**Table 4:** Pairwise and categorical statistics.

Name	Equation	Units	Perfect Score
Pearson's correlation coefficient (CC)	$\text{CC}=\frac{\frac{1}{n}\sum_{i=1}^{n}\left(Y_{i}-{\hat{Y}}_{i}\right)\left(Y_{0}-{\hat{Y}}_{o}\right)}{\sqrt{\sum_{i=1}^{n}{\left(Y_{i}-{\hat{Y}}_{i}\right)}^{2}}\sqrt{\sum_{i=1}^{n}{\left(Y_{0}-{\hat{Y}}_{o}\right)}^{2}}}$	-	1.0
Biweight midcorrelation (Bicor)	$\text{Bicor}=\;\frac{\zeta xy}{\sqrt{\zeta xx\;\zeta yy}}$	-	1.0
Percent bias (Pbias)	$\text{Pbias}=\;\frac{\sum_{i=1}^{n}\left(Y_{0}-Y_{i}\right)*100}{\sum_{i=1}^{n}\left(Y_{0}\right)}$	%	0.0
RMSE- observations standard deviation ratio (RSR)	$\text{RSR}=\frac{\sqrt{\sum_{i=1}^{n}{\left(Y_{0}-Y_{i}\right)}^{2}}}{\sqrt{\sum_{i=1}^{n}{\left(Y_{0}-{\hat{Y}}_{i}\right)}^{2}}}$	-	0.0

where $Y_{i}$=estimated index during i period, $Y_{o}$=observed index, N is the total number of observations, ζxx is the biweight midvariance of xx, ζyy is the biweight midvariance of yy, and ζxy is the biweight midcovariance of xx and yy.

**Fig. 4:**
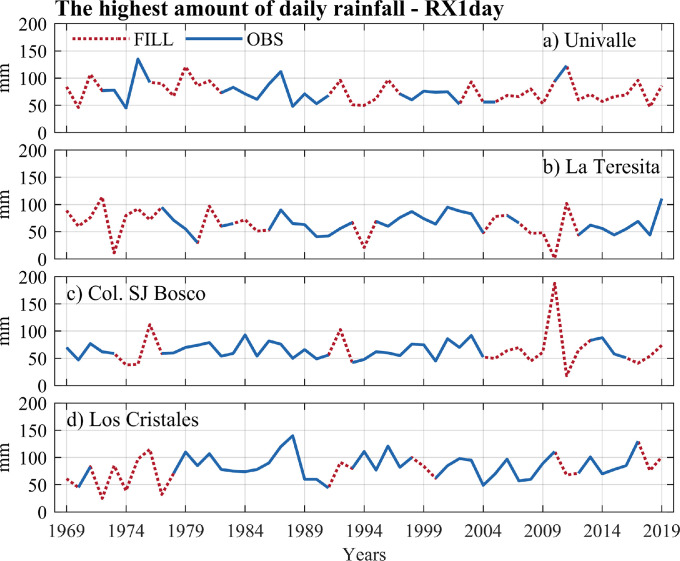
Annual observed time series of the highest of daily rainfall – RX1day index versus annual estimates for rainfall stations (a) Univalle, (b) La Teresita, (c) Col. SJ Bosco, and (d) Los Cristales.

**Fig. 5:**
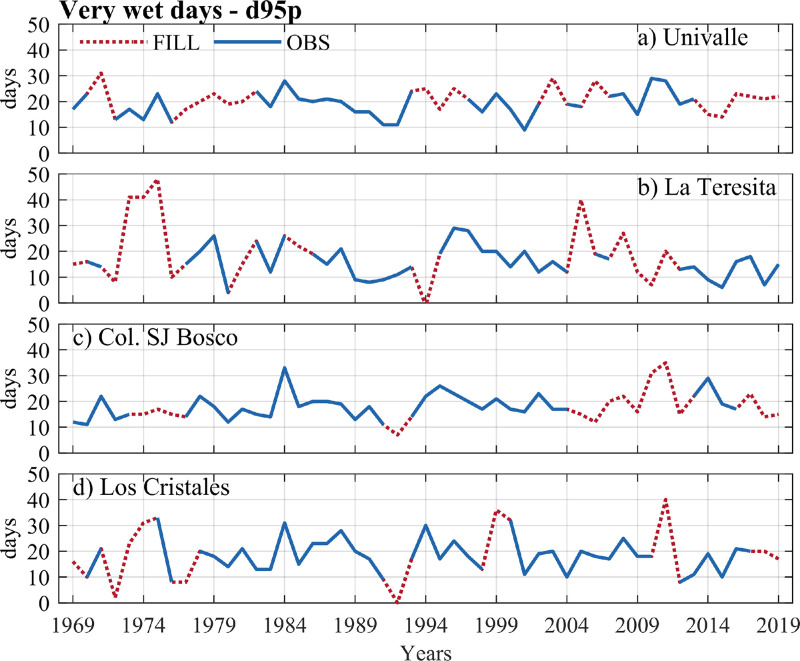
Annual observed time series of very wet days – d95p index versus annual estimates for rainfall stations a) Univalle, b) La Teresita, c) Col. SJ Bosco, and d) Los Cristales.

**Fig. 6:**
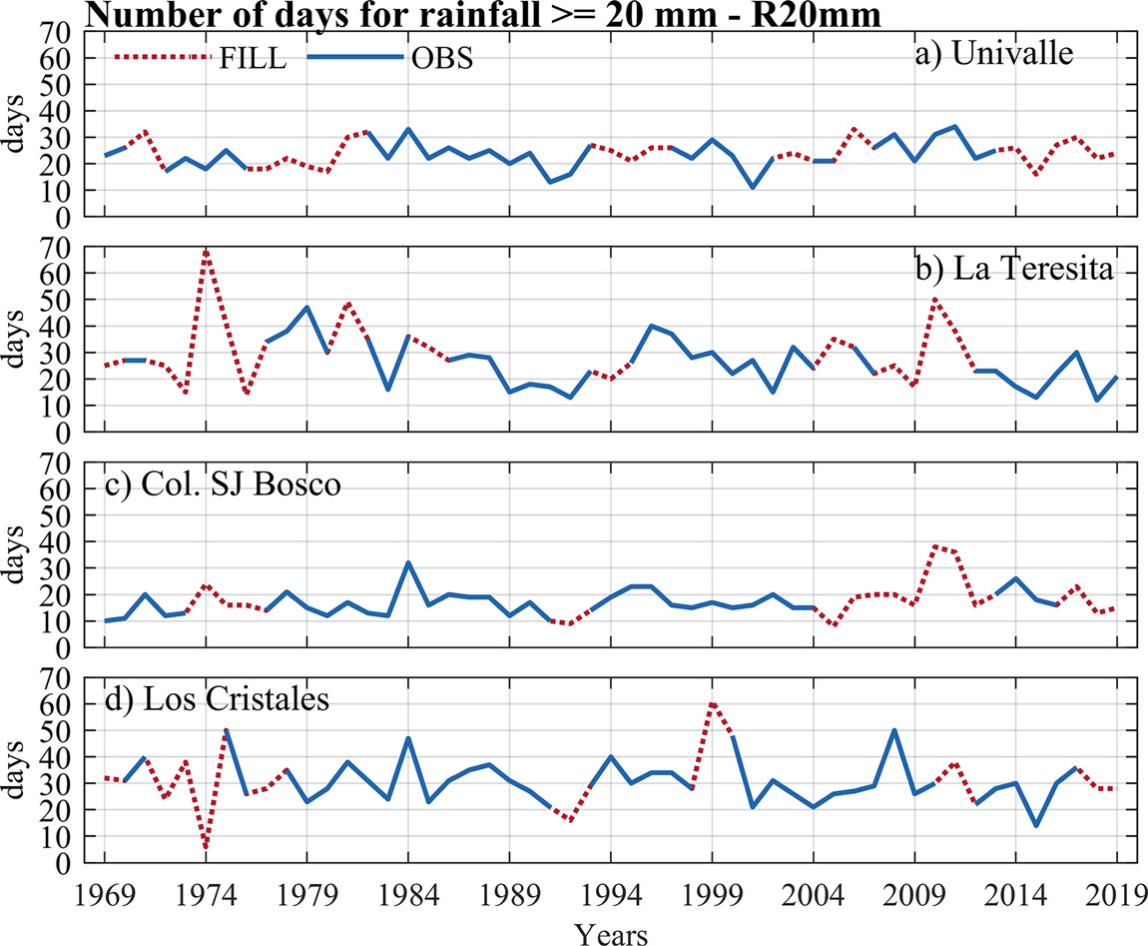
Annual observed time series of the number of days for rainfall >= 20 mm–R20 mm index versus annual estimates for rainfall stations a) Univalle, b) La Teresita, c) Col. SJ Bosco, and d) Los Cristales.

**Fig. 7:**
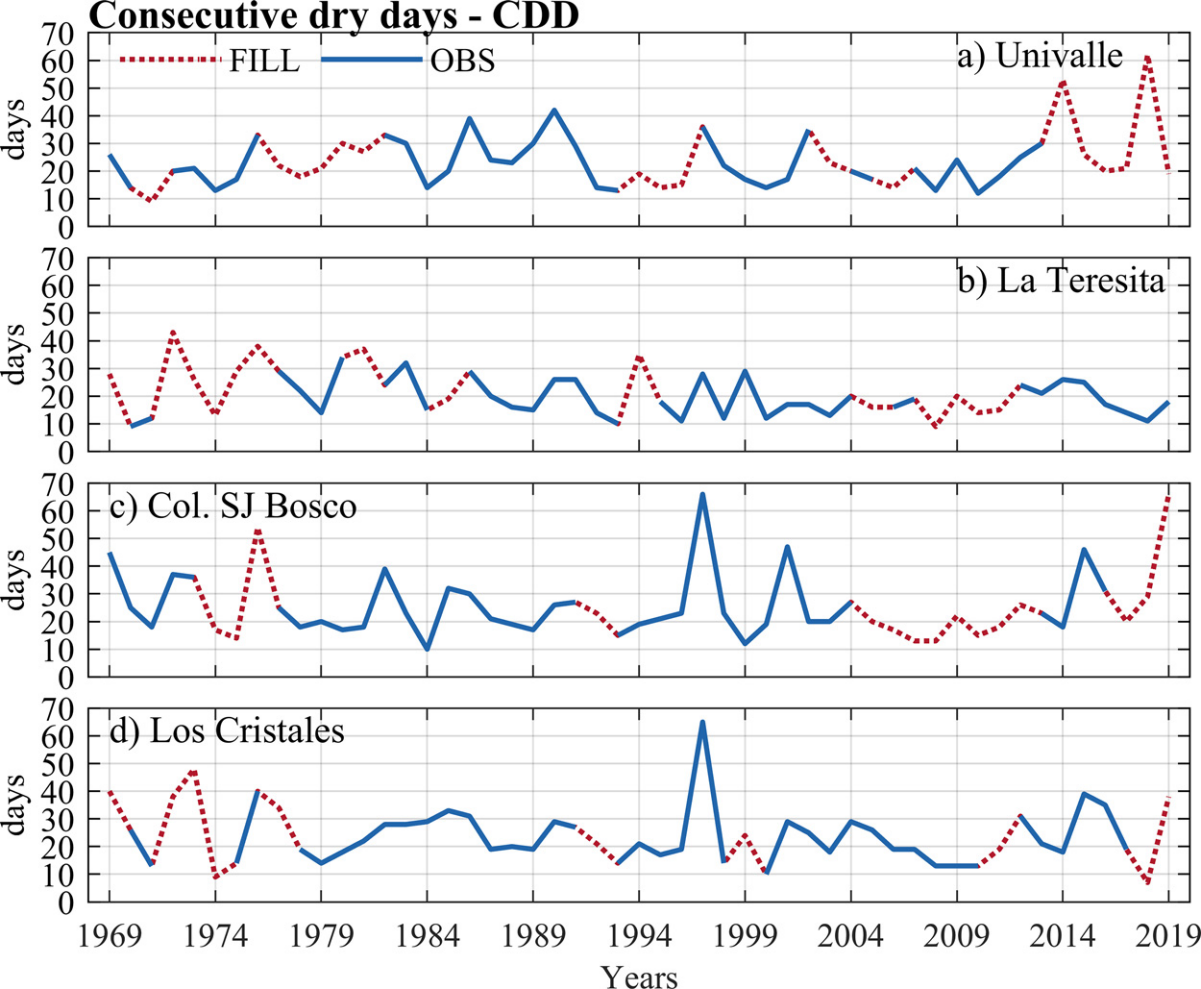
Annual observed time series of consecutive dry days – CDD index versus annual estimates for rainfall stations a) Univalle, b) La Teresita, c) Col. SJ Bosco, and d) Los Cristales

The data of the observed and estimated extreme rainfall indices are shown in Fig. 4, Fig. 5, Fig. 6, Fig. 7. Here, the annual series for two intensity indices (the highest amount of daily rainfall - RX1day, and very wet days - d95p), a frequency index (number of days for rainfall >= 20 mm–R20 mm), and a duration index (consecutive dry days - CDD) were presented. Four stations with a high percentage of missing data in extreme rainfall indices at the annual scale were plotted: Univalle (27%), La Teresita (24%), Col. SJ Bosco (22%), and Los Cristales (16%) (see Table 3). All the time series and graphs for the station's rainfall gauges at the annual/ seasonal scales indices are available in the Appendix.

## 2 Experimental Design, Materials and Methods

### 2.1 Study area description

The Metropolitan area of Cali (MAC) is located in southwestern Colombia, dynamically structured as a territorial and functional unit based on a physical environment. The MAC is the third-largest Metropolitan area in the country, with an estimated population of 3.9 million inhabitants in 2018 [2,3]. The MAC comprises Cali, Candelaria, Jamundí, Palmira, Florida, Pradera, and Yumbo, covering 3580 km^2^ in the geographic valley of the Cauca River, the most important basin in the country. The MAC's geomorphological, geological, and hydroclimatological characteristics promote hydrological hazards (e.g., floods and droughts), negatively affecting its economic and social development [4], [5], [6]. Therefore, the socio-economics of the region is affected, including losses in extensive territories dedicated to agriculture and livestock, urban and rural areas, partial or total destruction of infrastructure, and energy deficits [5,^7^,8].

### 2.2 Material and methods

#### 2.2.1 Rainfall indexes

The climate rainfall extreme indices were calculated using the ClimInd package (Available in http://etccdi.pacificclimate.org/software.shtml) [1]. The indicators are based on daily rainfall (RR) information (Table 2) of surface data (Table 1). Finally, 12 climate indices were selected related to rainfall extremes' intensity, frequency, and duration. The indexes are determined on the yearly and seasonal scale of each rainfall station.

#### 2.2.2 Missing data estimation

Considering that some stations exhibited more than 3 or 15 missing days at seasonal and annual scales, respectively. The auto-associative artificial neural network approach called NLPCA to estimate missing data in the time series of extreme rainfall indices were used. The methodology applied for the missing data estimation is based on the decoder, the second phase of the NLPCA, known as inverse NLPCA, a non-linear generalization of the standard Principal Component Analysis when they want to go back to the original representation. This algorithm was established by Scholz et al. [9,10], and was used to estimate missing data in the field of hydro-climatology by Miró et al. [11] and Canchala et al. [12].

The inverse NLPCA uses the reconstruction function $\Phi_{gen}:y\to \bar{x}$, performed by a feed-forward network. Eq. (1) shown the output $\hat{x}$ is dependent upon the input X and the ANN weights $w\epsilon W_{3},W_{4}$.

(1) $$\bar{x}=\Phi_{gen}\left(w,y\right)=W_{4}g\;\left(W_{3}y\right)$$

where the goal of the $\Phi_{gen}$ is to estimate a dataset $\bar{x}\;$approximate to the target data x by minimizing the squared error$∥x-{\bar{x}}^{2}∥$. More details about this technique are available in Scholz et al. [10,11], and the NLPCA toolbox used in this study is available at http://www.nlpca.org/matlab.html.

The missing data of the annual and seasonal extreme rainfall indices were obtained using the extreme rainfall indices described in Table 3 as the inputs and a [12,^11^,12] network topology (see Fig. 2).

## Ethics Statements

The authors agree that there are no ethics statements to be made.

## CRediT authorship contribution statement

**Camilo Ocampo-Marulanda:** Conceptualization, Methodology, Software, Data curation, Writing – original draft, Visualization, Investigation. **Wilmar L. Cerón:** Conceptualization, Methodology, Software, Writing – original draft, Writing – review & editing, Visualization, Investigation. **Alvaro Avila-Diaz:** Conceptualization, Methodology, Software, Data curation, Writing – original draft. **Teresita Canchala:** Supervision, Writing – original draft. **Wilfredo Alfonso-Morales:** Supervision, Writing – original draft, Writing – review & editing. **Mary T. Kayano:** Supervision, Writing – review & editing. **Roger R. Torres:** Supervision, Writing – review & editing.

## Declaration of Competing Interest

The authors declare that they have no known competing financial interests or personal relationships that could have appeared to influence the work reported in this paper.

## Appendix Supplementary materials

Image, application 1
